# Clear Reception: Elucidating the Binding Characteristics of Bisphenol A

**DOI:** 10.1289/ehp.116-a36b

**Published:** 2008-01

**Authors:** Julia R. Barrett

Several studies have indicated that bisphenol A (BPA), which is widely used in polycarbonate plastic and epoxy resin manufacturing, can disrupt normal endocrine function. Given that BPA’s estrogen receptor (ER) binding and interaction are 100–1,000 times weaker than those of endogenous hormones, researchers have hypothesized that BPA interacts with nuclear receptors other than ER. A recent study directly assayed BPA’s interaction with estrogen-related receptor-γ (ERR-γ), one of a family of “orphan” nuclear receptors—those for which natural ligands are unknown—and clarified the structural requirements that enable BPA to bind this receptor **[*EHP* 116:32–38; Okada et al.]**.

Earlier research by the same team demonstrated that BPA strongly binds ERR-γ. The research also showed that BPA preserves ERR-γ’s high constitutive activity. Receptors with constitutive activity trigger molecular events in the absence of a ligand; specific ligands known as inverse agonists can deactivate these receptors.

The authors emphasize the importance of this investigation by noting that ERR-γ is very strongly expressed in the mammalian brain during development and in the brain, lung, and other tissues in adults; unpublished results from this group show the highest expression in the placenta. It is possible that BPA’s binding ERR-γ could affect the receptor’s role by activating transcription at the wrong times.

Using tritium-labeled BPA, the researchers conducted the first saturation binding assay to precisely characterize how strongly BPA binds ERR-γ. They also ran competitive binding assays with BPA analogs and other industrial chemicals, including phenol derivatives, to identify which structural characteristics of the chemicals are critical for binding ERR-γ and maintaining its constitutive activity. They found specific, extremely high binding affinity of BPA for ERR-γ. BPA analogs varied in their ability to bind the receptor, and phenol derivatives were newly discovered to be potential candidates for ERR-γ–mediated endocrine disruption.

These findings raise the immediate question of whether reported BPA-related endocrine disruption might actually be mediated through ERR-γ rather than through ER. Additionally, the researchers stress the need to determine the normal physiologic roles of ERR-γ as well as the ways in which BPA might affect these roles. Given the strong expression of ERR-γ in the fetal brain and placenta, further information is especially urgent with regard to outcomes for newborns.

